# Bone Formation from Porcine Dental Germ Stem Cells on Surface Modified Polybutylene Succinate Scaffolds

**DOI:** 10.1155/2016/8792191

**Published:** 2016-06-23

**Authors:** Nergis Abay, Gorke Gurel Pekozer, Mustafa Ramazanoglu, Gamze Torun Kose

**Affiliations:** ^1^Genetics and Bioengineering Department, Yeditepe University, 34755 Istanbul, Turkey; ^2^Molecular Biology, Genetics and Biotechnology Department, Istanbul Technical University, 34469 Istanbul, Turkey; ^3^Center of Excellence in Biomaterials and Tissue Engineering, BIOMATEN, METU, 06800 Ankara, Turkey; ^4^Department of Oral Surgery, Faculty of Dentistry, Istanbul University, 34093 Istanbul, Turkey

## Abstract

Designing and providing a scaffold are very important for the cells in tissue engineering. Polybutylene succinate (PBS) has high potential as a scaffold for bone regeneration due to its capacity in cell proliferation and differentiation. Also, stem cells from 3rd molar tooth germs were favoured in this study due to their developmentally and replicatively immature nature. In this study, porcine dental germ stem cells (pDGSCs) seeded PBS scaffolds were used to investigate the effects of surface modification with fibronectin or laminin on these scaffolds to improve cell attachment, proliferation, and osteogenic differentiation for tissue engineering applications. The osteogenic potentials of pDGSCs on these modified and unmodified foams were examined to heal bone defects and the effects of fibronectin or laminin modified PBS scaffolds on pDGSC differentiation into bone were compared for the first time. For this study, MTS assay was used to assess the cytotoxic effects of modified and unmodified surfaces. For the characterization of pDGSCs, flow cytometry analysis was carried out. Besides, alkaline phosphatase (ALP) assay, von Kossa staining, real-time PCR, CM-Dil, and immunostaining were applied to analyze osteogenic potentials of pDGSCs. The results of these studies demonstrated that pDGSCs were differentiated into osteogenic cells on fibronectin modified PBS foams better than those on unmodified and laminin modified PBS foams.

## 1. Introduction

Bone tissue engineering research focuses on differentiation of different sources of stem cells into bone cells on novel biocompatible biomaterials [1]. Also cell, scaffold, and biosignaling molecules with biomaterials have been used to form suitable cellular environments for tissue regeneration [2].

Scaffolds are the nonliving component of tissue engineering. Polybutylene succinate (PBS) is a novel biodegradable aliphatic polyester and can be used in bone tissue engineering applications because of its good mechanical properties, adjustable degradation rate, and nontoxic degradation products for the healing of bone defects. In addition to that, the use of PBS as a scaffolding material for bone repair is advantageous due to its processability. However, the surface modification is still required for the improvement of the biocompatibility and bioactivity of PBS scaffolds [3].

Surface modification by surface coating provides a way to conserve the mechanical properties of materials and to improve the surface biocompatibility of scaffolds. Most of the extracellular matrix (ECM) proteins, such as fibronectin, laminin, vitronectin, and collagen, have a sequence of amino acids like arginine–glycine–aspartic acid (RGD) which can be recognized by cells. Integrin-mediated binding of cells to those bioactive surfaces supports cell attachment, proliferation, and differentiation. Integrin-binding mechanism supplies communication of cells with noncellular surroundings. Besides, prolonged proliferation and survival can be observed by the coating of the surface of the scaffold with protein molecule. Particularly, either fibronectin or collagen type I treated surfaces exhibit both mineralization and the presence of bone formation better than laminin treated surfaces [4, 5].

In the bone tissue engineering field, bone marrow is the most widely used source of mesenchymal stem cells (MSCs) [6]. However, bone marrow collection from a patient is an invasive procedure. Thus, scientists focused on finding new sources of mesenchymal stem cells which require minimally invasive collection procedures. Since the first isolation and characterization of stem cells from dental pulp in 2000 [7], dental tissues gained attention as rich mesenchymal stem cell sources due to accessibility and multilineage differentiation capacity [8].

Dental stem cells (DSCs) that are an attractive alternative source of MSCs easily differentiated into osteo-, adipo-, and neurogenic cells [9] are comprised of dental pulp stem cells (DPSCs), dental follicle stem cells (DFSCs), stem cells from exfoliated deciduous teeth (SHED), periodontal ligament stem cells (PDLSCs), stem cells from immature dental tissues such as apical papilla (SCAP), and dental germs which contain follicle and surrounding tissues (DGSCs) [10]. They are derived from neural crest and contain both ectodermal and mesenchymal components. Isolation of dental germ stem cells (DGSCs) from immature teeth such as third molars (wisdom teeth) has a definite advantage because of their ability to come from an organ. They are known as a source of more developmentally immature stem cells that have increased proliferation and differentiation potentials [9].

Domestic pig is preferred as an experimental model to isolate DGSCs due to its anatomical, physiological, and metabolic similarities with humans. Also, the diphyodont and heterodont dentition of the pig resemble that of humans which makes it a good candidate to study tooth morphogenesis and dental stem cell-mediated tissue engineering. In this study, bone regeneration potential of DGSCs on unmodified and fibronectin and laminin modified PBS scaffolds was investigated for the first time in the literature to treat the critical size bone defects.

## 2. Materials and Methods

### 2.1. Preparation of Poly(1,4-butylene succinate) Scaffolds

Poly(1,4-butylene succinate), extended with 1,6-diisocyanatohexane (*M*_*n*_ = 5.0 × 10^5^, *M*_*w*_/*M*_*n*_ = 2.7) (Sigma-Aldrich Corporation, Germany) solution (4%), was prepared by using chloroform (Sigma-Aldrich Corporation, Germany) as a solvent. This solution was sonicated on ice for 2 h and then transferred into an Erlenmeyer flask. PBS scaffolds were prepared by solvent casting/particulate leaching technique using 300–500 *μ*m salt particles for the leaching. In order to obtain a porous scaffold, salt was removed by leaving the scaffold overnight in distilled water and changing the distilled water 3 times until all the salt was removed. Porous polymers were then frozen at 80°C and freeze-dried for 48 h. Scaffolds were prepared by cutting into circular pieces of 1 × 1 cm^2^.

### 2.2. Characterization of PBS Scaffolds by Scanning Electron Microscopy

PBS scaffolds were coated with gold by Sputter Coater (BAL-TEC SCD 005, Germany) and samples were analyzed with Scanning Electron Microscope (Carl Zeiss EVO, Germany).

### 2.3. Degradation of PBS Scaffolds

For this study, PBS scaffolds that were weighed were put into 20 mL of 0.09% sodium azide in physiological isotonic saline solution (0.0095 M PO_4_, pH: 7.32) in the sterile centrifuge tubes and they were placed into the shaking water bath at 37°C. After 7th, 15th, 30th, 60th, and 120th days, pH of physiological isotonic saline solution was measured to observe the decrease of pH values due to the degradation of PBS scaffolds. After that, PBS scaffolds were lyophilized and their weights were measured. According to that, the rate of degradation of PBS scaffolds was analyzed.

### 2.4. Surface Modification of PBS Scaffolds with Adhesion Proteins

PBS foams were placed into the 24-well cell culture plates and sterilized in 70% ethanol for 2 h at 4°C. After they were washed with physiological saline solution, the foams were dried under the laminar flow cabinet (Telstar, Bio-II-A, Spain). PBS foams were coated with either fibronectin or laminin solution (50 *μ*g/mL, 500 *μ*L) (Roche, USA) by incubating them in CO_2_ incubator (37°C, 5% CO_2_) (Thermo Scientific, USA) for 2 h. When the incubation was completed, foams were washed with physiological saline solution to remove excess protein. Unmodified foams were used as control.

### 2.5. *In Vitro* Cell Culture Studies

#### 2.5.1. Isolation and Culturing of pDGSCs

The cells were isolated by explant culture of tooth germs excised from 6-month-old domestic pigs under anesthesia and aseptic conditions approved by Yeditepe University Animal Research Local Ethics Committee (YÜDHEK). Tooth germ tissues were minced with a sterile scalpel and allowed cells to migrate from the tissue and adhere to tissue culture plate. When the cells reached confluency after 1 week, they were frozen for further use. For this study, cells that were previously frozen and kept in liquid nitrogen were thawed and expanded in an incubator at 37°C, in 5% CO_2_ and 90% humidity. The medium was changed every other day until confluency was reached.

#### 2.5.2. Characterization of pDGSCs by Flow Cytometry

pDGSCs (P1) were trypsinized and counted with hemacytometer (Hausser Bright-Line, USA). Cells (5 × 10^5^) for each antibody and negative control were taken into FACS tubes. They were incubated with the antibodies for CD34 (BD Pharmingen, Philippines), CD45 (BD Pharmingen, Philippines), CD105 (Abcam, USA), CD90 (BD Pharmingen, Philippines), and CD44 (Abcam, USA) cell surface markers at room temperature for 1 h. Cells were washed with physiological saline solution and centrifuged at 2,200 rpm for 5 min to remove excess antibodies. After centrifugation, they were resuspended in 400 *μ*L physiological saline solution and samples were analyzed in flow cytometer (FACSCalibur, Becton Dickinson, USA).

#### 2.5.3. Cell Seeding

Cells (30,000/foam) were seeded onto the sterilized unmodified, fibronectin modified, and laminin modified PBS foams. Cell seeded foams were incubated in a CO_2_ incubator at 37°C, in 5% CO_2_ and 90% humidity throughout 20 days. At day 3, osteogenic medium (Dulbecco's Modified Eagle Medium (DMEM: 4.5 g/liter glucose)) (Gibco Invitrogen, USA) supplemented with 10% fetal bovine serum (FBS) (Gibco-Invitrogen, USA), 100 units/mL Penicillin-Streptomycin-Amphotericin (PSA) (Lonza, Switzerland), 50 *μ*M ascorbic acid (AppliChem, Germany), 100 nM dexamethasone (AppliChem, Germany), and 10 mM *β*-glycerophosphate (AppliChem, Germany) was added into the wells and it was changed twice a week. All tests were carried out in triplicate throughout the whole study.

#### 2.5.4. Determination of Cell Viability on Modified and Unmodified PBS Scaffolds by MTS Assay

The CellTiter 96® Aqueous One Solution Cell Proliferation Assay (MTS) (Promega, USA) was applied to the cell seeded modified and unmodified foams at 3, 4, and 10 days of incubation.

For this assay, first of all, cell seeded PBS foams were transferred to new 24-well plate and washed with physiological saline solution to remove media which were left overnight before. MTS working solution (MTS: media mixture (1 : 5), 500 *μ*L) was added onto each cell seeded foam in 24-well plate and incubated for 2 h at 37°C in a CO_2_ incubator. After the incubation, 200 *μ*L of solution from each well was transferred into a 96-well plate. Absorbances were measured at 490 nm by using Elisa Plate Reader (Bio-Tek, Elx800, USA). Absorbance values were then converted to cell numbers according to a calibration curve that was constructed by using known cell numbers.

#### 2.5.5. Alkaline Phosphatase Assay

Alkaline phosphatase (ALP) (Randox, UK) assay was applied to pDGSCs on modified and unmodified PBS scaffolds at 4, 10, and 20 days of incubation. In this assay, the absorbance was measured at 405 nm for time points of 0, 2, 4, 6, 8, 10, 12, and 14 min by Elisa Plate Reader. After the measurement of absorbance values, the slope of the absorbance versus time plot was used to calculate the alkaline phosphatase activity per min.

After the measurement of absorbance values, DNA was isolated from each sample and their DNA concentrations were measured using NanoDrop (Implen NanoPhotometer® P-Class, USA). To get normalized values, absorbance values were divided into DNA concentrations of each sample and the normalized ALP graph was constructed.

#### 2.5.6. Determination of Mineralization by von Kossa Staining

von Kossa staining (Sigma-Aldrich Corporation, Germany) procedure was used to determine the extent of minerals deposited on PBS scaffolds. Mineralization of pDGSCs seeded on unmodified and fibronectin and laminin modified PBS scaffolds and TCP at the end of 10 and 20 days of incubation was evaluated. In this method, silver nitrate solution (500 *μ*L) (Sigma-Aldrich Corporation, Germany) was added onto PBS scaffolds and the plate was exposed to ultraviolet light for 30 min. Positively charged silver ions reacted with negatively charged phosphates and carbonates in calcium deposits and were then reduced to black metallic silver by UV light. After that, the scaffolds were washed with physiological saline solution and the reaction was stopped with the addition of 500 *μ*L of 5% sodium thiosulfate (Sigma-Aldrich Corporation, Germany). After scaffolds were washed with physiological saline solution, their images were obtained by inverted microscope (Nikon Eclipse TC 100, USA). Ultimately, cells were stained with nuclear fast red to label the nucleus and cytoplasm of cells to make cells apparently visible for brightfield microscopy.

#### 2.5.7. Immunostaining of PBS Scaffolds

Cells on the scaffolds were fixed using 3.7% formalin containing 0.001% Tween® 20 (AppliChem, Germany) for 30 min at room temperature after 10 and 20 days of incubation. After fixation, samples were incubated with 3% FBS in physiological saline solution at room temperature for 10 min to prevent unspecific binding of the dyes used for confocal microscopy study.

Alexa Fluor 546 Phalloidin (Molecular Probes, Invitrogen, USA) dye was mixed with 1.5% FBS in physiological saline solution with a ratio of 2 : 100, and this mixture was added onto each sample. Scaffolds were incubated with Phalloidin solution for 1 h at 37°C and washed with physiological saline solution to remove excess Phalloidin.

For collagen type I staining (Millipore, USA), the primary antibody of collagen type I was mixed with 1.5% FBS in physiological saline solution with a ratio of 1 : 100. Solution of collagen type I primary antibody was added onto the foams and the samples were incubated at 37°C for 1 h. Then, samples were washed with physiological saline solution. Secondary antibody of collagen type I was mixed with physiological saline solution with a ratio of 1 : 100 and added onto the samples. Samples were incubated at 37°C for 1 h. After the incubation, samples were washed with physiological saline solution.

After the staining of the samples with Phalloidin or collagen type I, all samples were double stained with TO-PRO solution. For that purpose, TO-PRO-3 Iodide (Molecular Probes, Invitrogen, USA) dye was diluted with a ratio of 2 : 100 using 1.5% FBS in PBS solution. TO-PRO dye was added to the wells and samples were incubated at 37°C for 15 min. After the incubation, TO-PRO dye was removed, and samples were washed with physiological saline solution. Finally, all samples were cured with Prolong Gold antifade reagent (Invitrogen, USA) at 4°C overnight. The foams were observed using confocal microscope (Leica, Germany).

#### 2.5.8. CellTracker CM-Dil Staining of PBS Scaffolds

CM-Dil stock solution (1 mg/mL) (Invitrogen, USA) was prepared from 50 *μ*g CM-Dil powder. CM-Dil solution was used at 2 *μ*M concentration to stain the cells at 37°C for 10 min. Cells were washed and then seeded onto the unmodified, fibronectin modified, and laminin modified PBS scaffolds. As a control, cells were also seeded onto the surface of tissue culture plates.

#### 2.5.9. Real-Time PCR Analysis

Expressions of bone-specific genes such as osteopontin, osteocalcin, ALP, Runx2, and type I collagen were determined by real-time PCR analysis. Briefly, after 10 and 20 days of incubation period, total RNA was extracted from the cells on the scaffolds using RNA extraction kit (Thermo Scientific, USA). Total mRNA was reverse-transcribed into cDNA using the Sensiscript RT Kit (Qiagen, Germany). Real-time PCR analysis was performed using Maxima SYBR Green Master Mix (Thermo Scientific, USA) and the reaction was carried out in Bio-Rad CFX96 Touch*™* Real-Time PCR Detection System for cDNAs of osteogenic genes and beta-actin (*β*-actin) as housekeeping gene. *β*-Actin mRNA was used as an internal control for normalization. Sequences of primers for both osteogenic and housekeeping mRNAs are listed in Table 1.

## 3. Statistical Analysis

Statistical significance was assessed using two-tailed *t*-test on Excel. Differences were considered as statistically significant when *p* ≤ 0.05 and *p* ≤ 0.1.

## 4. Results

### 4.1. Characterization of Scaffolds by Scanning Electron Microscopy

**Figure 1:**
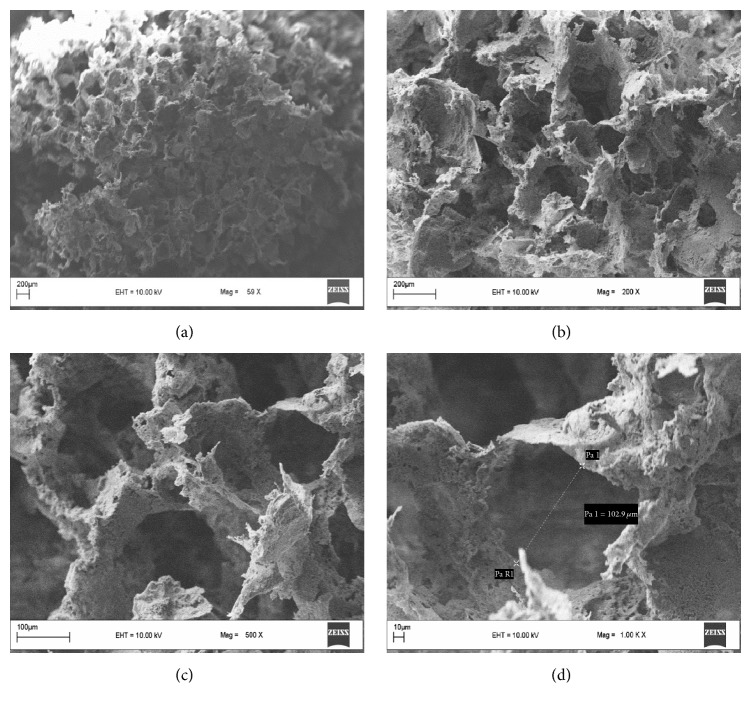
SEM images of PBS scaffolds: (a) 59x, (b) 200x, (c) 500x, and (d) 1,000x objectives.

PBS scaffolds were prepared by salt leaching technique and observed by SEM to investigate the surface characteristics and porosity. In Figure 1, the structural details were observed and the pore sizes were measured by Scion Image Analyzer. The average pore size was found around 100 *μ*m. Appropriate pore size provides an advantage for cells to penetrate inside the scaffolds to support cell growth and development homogeneously throughout the scaffold. Also, porosity and pore size are important for nutrient and oxygen diffusion to the deep inside the scaffolds. According to the SEM images, the surfaces of PBS scaffolds were rough and provided cell attachment (Figure 1).

### 4.2. Degradation of PBS Scaffolds

**Figure 2:**
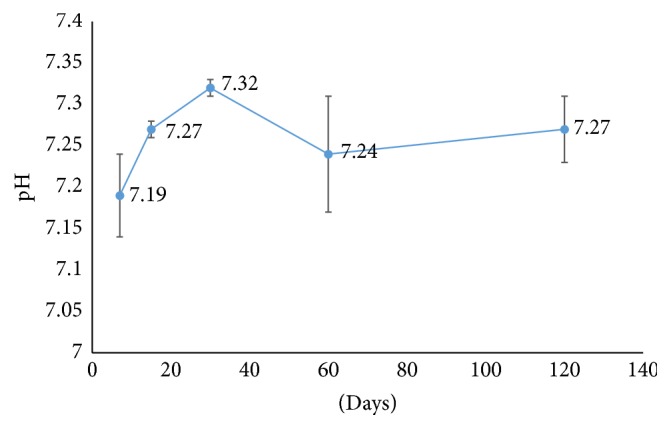
Degradation of PBS scaffolds with respect to pH throughout 120 days of incubation.

In the first part of the experiment, the changes of pH values of PBS scaffolds were obtained on 7th, 15th, 30th, 60th, and 120th days of incubation (Figure 2). The pH level increased slightly during the first 30 days. After 30 days, decrease in pH level was observed. The changes in the pH were statistically not significant. It was observed that throughout 120 days of incubation there was no drastic change in the pH of the environment which might harm the cells.

**Figure 3:**
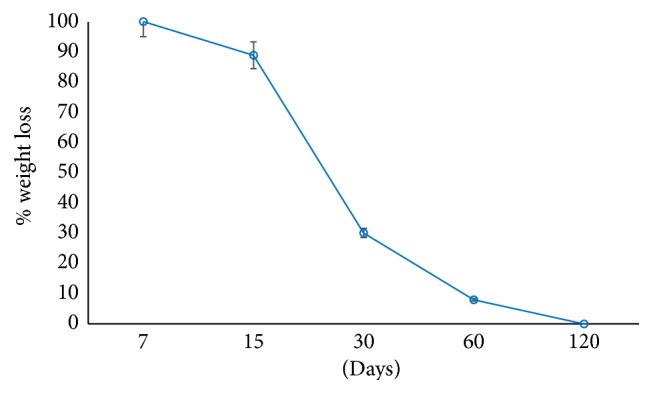
Degradation of PBS scaffolds with respect to weight during 120 days of incubation.

In the second part of degradation experiment, % weight loss of PBS scaffolds throughout 120 days of incubation was investigated. It was observed that after 15 days % weight loss was approximately 11.2, and then it reached 69.93 after 30 days of incubation. At the end of 120 days of incubation, PBS scaffolds completely degraded (Figure 3).

### 4.3. Characterization of pDGSCs by Flow Cytometry Analysis

**Figure 4:**
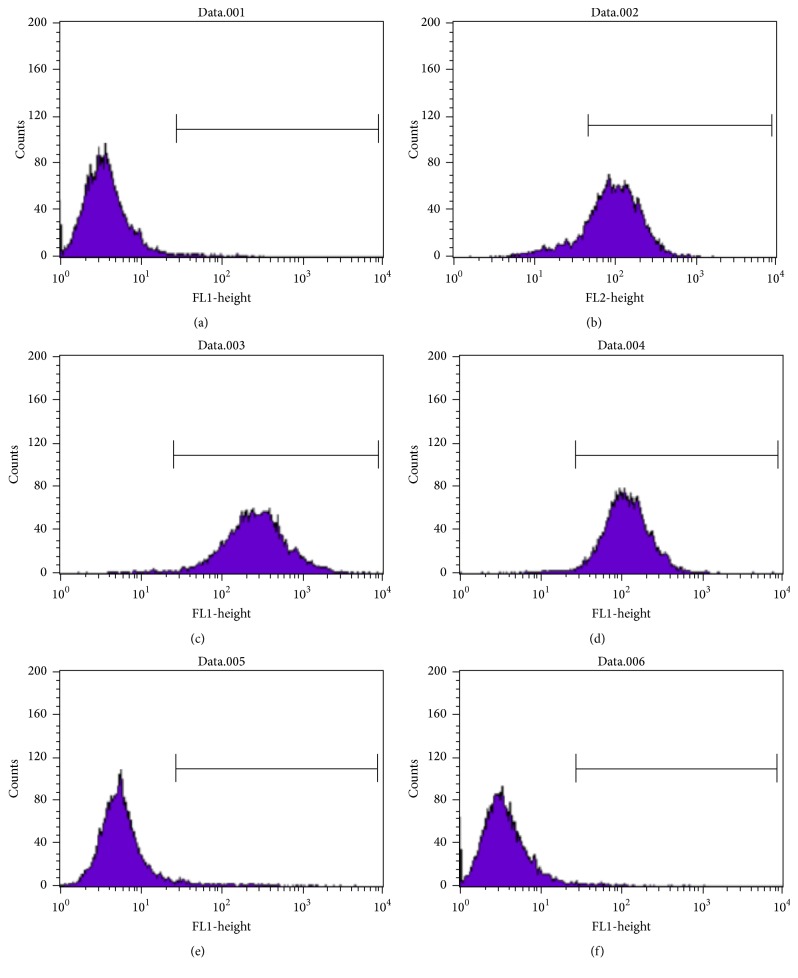
Flow cytometry histogram of pDGSCs with a label by FACSCalibur: (a) only cells without antibody, (b) CD105, (c) CD90, (d) CD44, (e) CD45, and (f) CD34.

The mesenchymal stem cell properties of pDGSCs were investigated by tagging mesenchymal stem cell related (CD105, CD90, and CD44) and hematopoietic stem cell (CD45 and CD34) related cell surface markers. Cells were negative for CD45 and CD34 hematopoietic stem cell markers and positive for CD105, CD90, and CD44 mesenchymal stem cell markers (Figure 4). Percentage of positively marked cells for each antibody is listed in Table 2. Cell surface marker expression shows that isolated cells were mesenchymal stem cells.

### 4.4. Cell Viability on PBS Scaffolds

**Figure 5:**
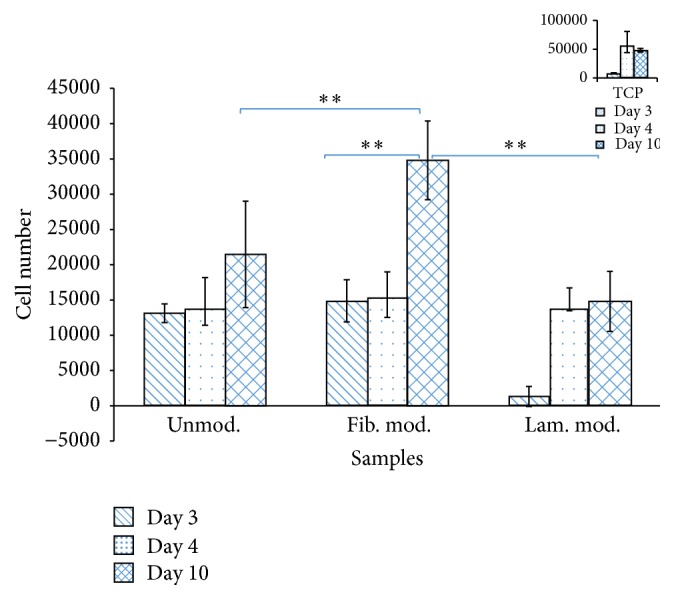
Cell proliferation on surface modified and unmodified foams after 3, 4, and 10 days of incubation. Initial cell seeding was 3 × 10^4^ cells/sample (^*∗∗*^ *p* ≤ 0.05). Indent on the top right corner shows the cell proliferation on tissue culture plate.

The effect of fibronectin and laminin modification of PBS scaffolds on the proliferation of pDGSCs was investigated by MTS cell proliferation assay after 3, 4, and 10 days of incubation.  Figure 5 showed that cell numbers increased in both modified and unmodified scaffolds through 10 days of incubation.

The highest cell number at all time points was found on TCP. However, at the end of 10-day incubation period, decrease in cell number was observed on TCP due to the cell death related to lack of available space. At the end of 3 days of incubation period, cell numbers were found as 1.5 × 10^4^ on fibronectin modified scaffold, 1.3 × 10^4^ on laminin modified scaffold, and 1.2 × 10^4^ on unmodified scaffold. After 10 days of incubation, cell number on fibronectin modified scaffold (3.5 × 10^4^ cells/foam) was significantly (*p* ≤ 0.05) higher than the cell numbers on both laminin modified (1.4 × 10^4^ cells/foam) and unmodified scaffolds (2.1 × 10^4^ cells/foam).

### 4.5. Alkaline Phosphatase Assay

Alkaline phosphatase activity of pDGSCs on PBS foams was investigated at 4, 10, and 20 days. Alkaline phosphatase assay was used to measure the conversion of p-nitrophenyl phosphate to p-nitrophenol in the presence of alkaline phosphatase at 405 nm.

**Figure 6:**
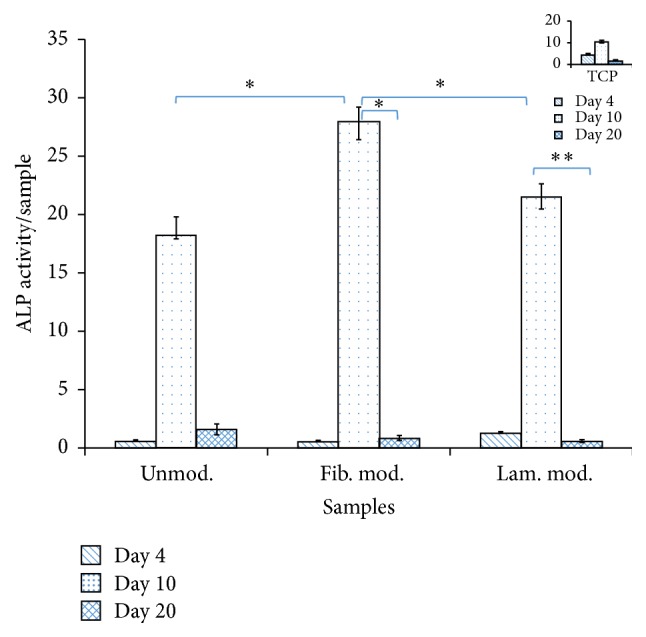
Normalized ALP activity of pDGSCs on modified and unmodified scaffolds throughout 20 days of incubation (^*∗*^ *p* ≤ 0.1 and ^*∗∗*^ *p* ≤ 0.05). Indent on the top right corner shows ALP activity of cells seeded on tissue culture plate.

It was observed from Figure 6 that in all samples ALP expression was similar during early stage (day 4) of differentiation. At 10 days of incubation, approximately 1.3-fold difference in the ALP activity was observed when unmodified and fibronectin modified scaffolds were compared. ALP level was significantly (*p* ≤ 0.1) higher in fibronectin modified samples than that in unmodified and laminin modified ones. It was demonstrated that the differentiation of pDGSCs was higher on fibronectin modified samples than the others. Then, 12-fold significant decrease of alkaline phosphatase activity was seen from 10 to 20 days of incubation in unmodified scaffolds. Also, significant (35-fold) decrease from day 10 to day 20 was seen in modified scaffolds (*p* ≤ 0.1 and *p* ≤ 0.05).

### 4.6. von Kossa Staining

**Figure 7:**
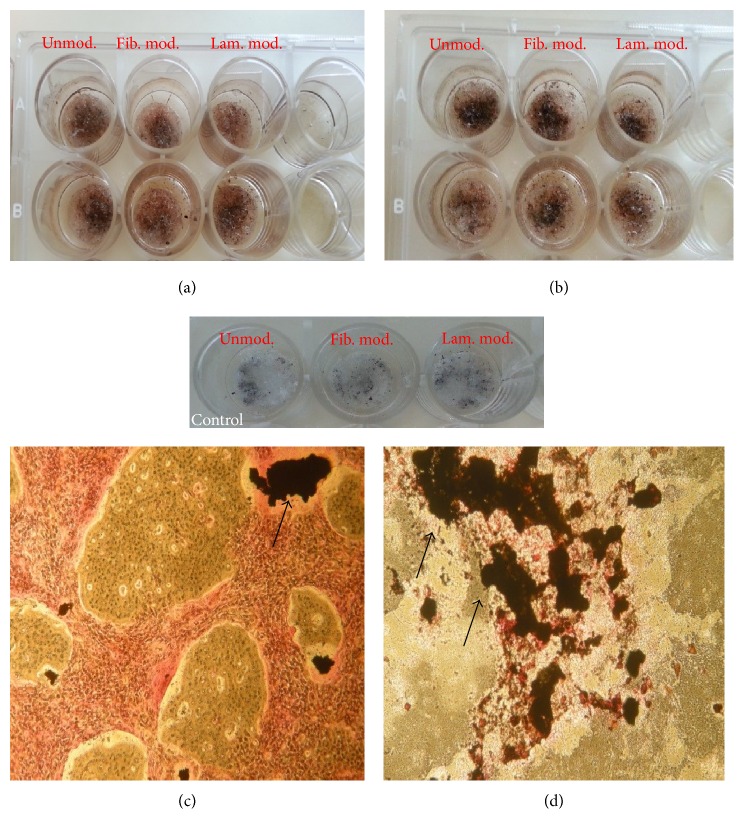
Mineralization of pDGSCs seeded on (a, b) unmodified and fibronectin and laminin modified PBS scaffolds; (c, d) TCP (10x) at the end of (a, c) 10 and (b, d) 20 days of incubation. Arrows indicate mineralized nodules.

von Kossa staining was used for the observation of mineralization on unmodified, fibronectin modified, and laminin modified PBS scaffolds after 10 and 20 days of incubation. Mineralization of pDGSCs seeded on TCP was observed at the end of 10 and 20 days of incubation (Figures 7(c) and 7(d)). The brown regions demonstrated the presence of mineralized nodules on TCP. After 10 days of incubation, red regions that were the indication of the presence of cells appeared more than brown regions. However, red areas reduced at the end of 20 days of incubation.

Mineralization is known as a late marker of osteoblast differentiation. In this study, mineralization was observed at high levels on unmodified foams at the end of 10 days of incubation period compared to either laminin or fibronectin modified PBS scaffolds (Figure 7(a)). At this time point, the extent of mineralization was not too much on fibronectin modified scaffold whereas more mineralization was observed on the laminin modified scaffold. However, after 20 days of incubation, mineralization on the fibronectin modified scaffold was found to be higher than the others (Figure 7(b)).

### 4.7. Immunostaining with Confocal Microscopy

Immunostaining with confocal microscopy studies was conducted for the investigation of the cell-to-cell and cell-to-scaffold relation after 10 and 20 days of incubation periods. Alexa Fluor 546 Phalloidin and TO-PRO-3 Iodide were used to stain the cells.

**Figure 8:**
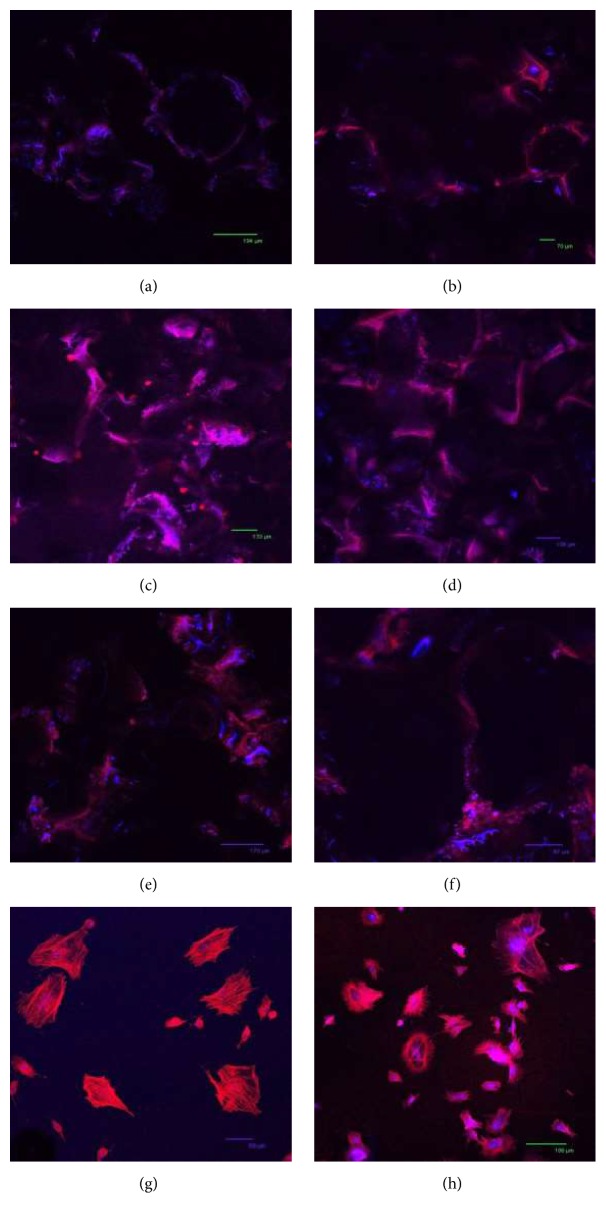
Confocal microscopy images of cells seeded on (a, b) unmodified; (c, d) fibronectin modified; and (e, f) laminin modified PBS scaffolds; (g, h) TCP after (a, c, e, g) 10 days and (b, d, f, h) 20 days of incubation. Red stains show actin filaments in the cytoskeleton of the cells stained with Alexa Fluor® 546 Phalloidin and blue stains show the nucleus of the cells stained with TO-PRO-3® Iodide. Scale bars (a) 194 *μ*m, (b) 70 *μ*m, (c) 133 *μ*m, (d) 106 *μ*m, (e) 170 *μ*m, (f) 97 *μ*m, (g) 69 *μ*m, and (h) 186 *μ*m.

When the microscope images of cells stained with Phalloidin were analyzed, the attachment of cells onto the surface of scaffolds was observed (Figure 8). At 10 days of incubation, cell-to-cell interaction on the unmodified and fibronectin and laminin modified PBS scaffolds was observed (Figures 8(a), 8(c), and 8(e)). It was also observed that cells mostly attached and proliferated around the pores of the foam where nutrient and oxygen diffusion is easier. Cell morphology and organization of actin filaments were investigated more clearly on TCP (Figures 8(g) and 8(h)). The amount of cell proliferation decreased from day 10 to day 20 on both TCP (Figure 8(h)) and PBS scaffolds (Figures 8(b), 8(d), and 8(f)).

**Figure 9:**
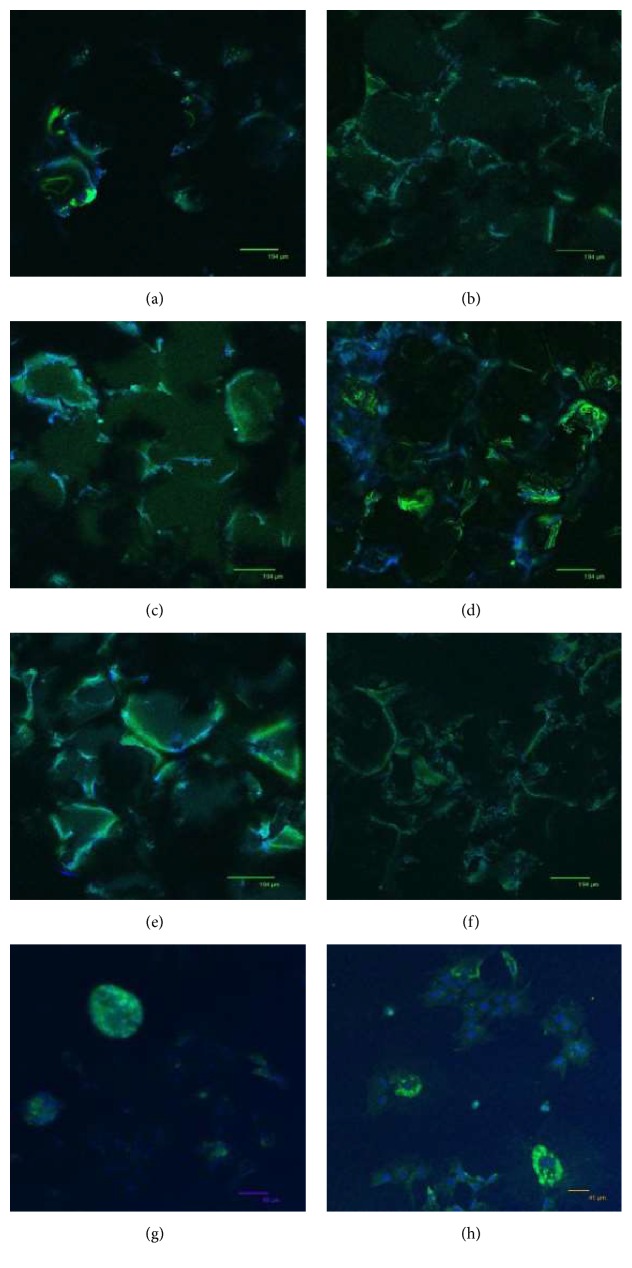
Confocal microscopy images of cells seeded on (a, b) unmodified; (c, d) fibronectin modified; and (e, f) laminin modified PBS scaffolds; (g, h) TCP after (a, c, e, g) 10 days and (b, d, f, h) 20 days of incubation. Green stains show the synthesis of collagen in the cytoskeleton of the cells stained with collagen type I and blue stains show the nucleus of the cells stained with TO-PRO-3 Iodide. Scale bars (a, b, c, d, e, f) 194 *μ*m, (g) 85 *μ*m, and (h) 41 *μ*m.

In order to observe ECM synthesis by the cells seeded on modified and unmodified foams and on glass slides, type I collagen fibers were tagged with primary and secondary antibodies. Collagen type I fibers are the most important component of the organic matrix, as well as an important determinant of bone differentiation. When the images of cell seeded unmodified, fibronectin modified, and laminin modified scaffolds that were stained with collagen type I were observed (Figures 9(a) and 9(e)), the increase in the intensity of the green staining showing the synthesis of collagen could be seen from 10 to 20 days of incubation period, especially in fibronectin modified PBS scaffolds (Figures 9(c) and 9(d)). These images show induced bone differentiation, especially on fibronectin modified samples at 20 days of incubation. Also, throughout 20 days of incubation on TCP, the increase in collagen type I was observed around the cells (Figures 9(g) and 9(h)).

### 4.8. CellTracker CM-Dil Staining

**Figure 10:**
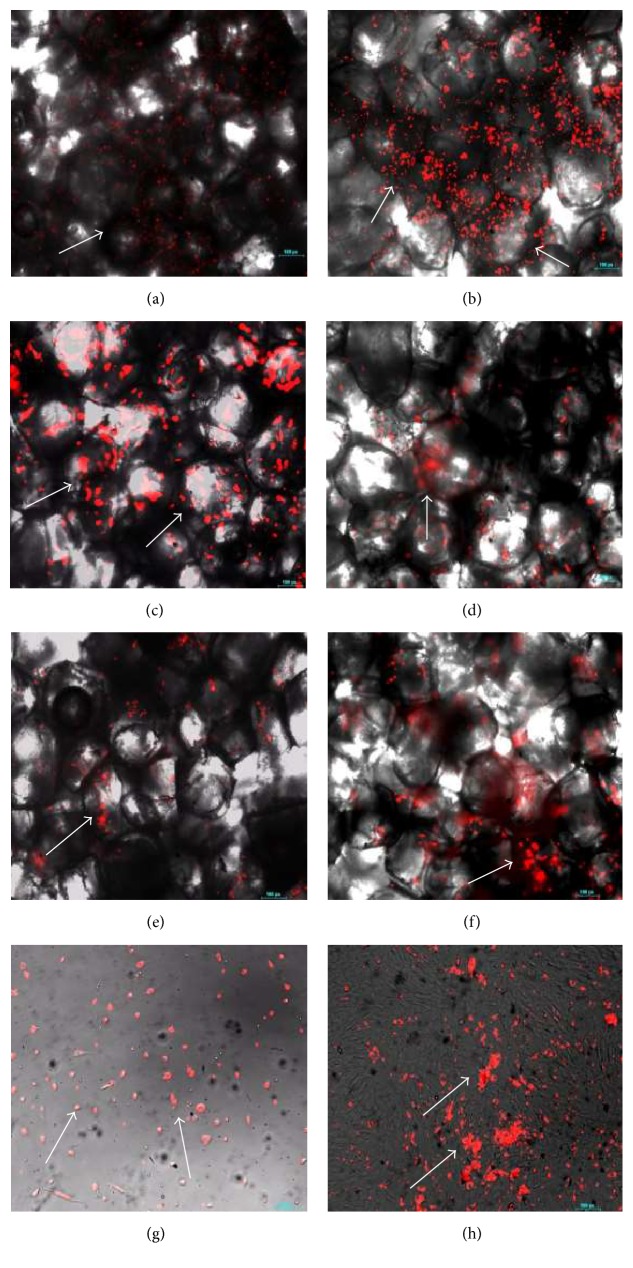
The images of viable cells stained with CellTracker*™* CM-Dil on (a, b) unmodified; (c, d) fibronectin modified; and (e, f) laminin modified PBS scaffolds; (g, h) TCP after 1 day (a, c, e, g) and 7 days (b, d, f, h) of incubation. Scale bars show 100 *µ*m. Arrows indicate viable cells.

CM-Dil is a fluorescent dye that has a thiol-reactive chloromethyl moiety which can bind to cellular thiols. By using this technique, viable cells can be stained without any fixation step, unlike other fluorescent dyes. In this way, the migration of viable cells and the increase of their cell number can be observed easily under the fluorescent microscope. The images showed that cells seeded on TCP, modified, and unmodified scaffolds still maintained their viability (Figure 10).

When the images were investigated, high amount of viable cells was seen on the unmodified PBS scaffolds at the end of 1 day of incubation (Figure 10(a)). Throughout 7 days of incubation, the increase in cell number was observed on the same unmodified PBS scaffold (Figure 10(b)). However, on either fibronectin or laminin modified scaffold, once cells were attached onto the surfaces, they migrate inside the pores and proliferate there from 1 to 7 days of incubation, especially on fibronectin modified scaffold (Figures 10(c) and 10(d)). Also, the cells were found around the pores, especially on fibronectin modified scaffolds. On TCP flasks, the highest cell number was observed throughout 7 days of incubation (Figures 10(g) and 10(h)). However, after 7 days of incubation period (data not shown), decrease in cell number was observed on TCP as it was expected due to the lack of available space for cells to grow more and the detachment of cells from the surface of TCP was seen.

### 4.9. Real-Time PCR

**Figure 11:**
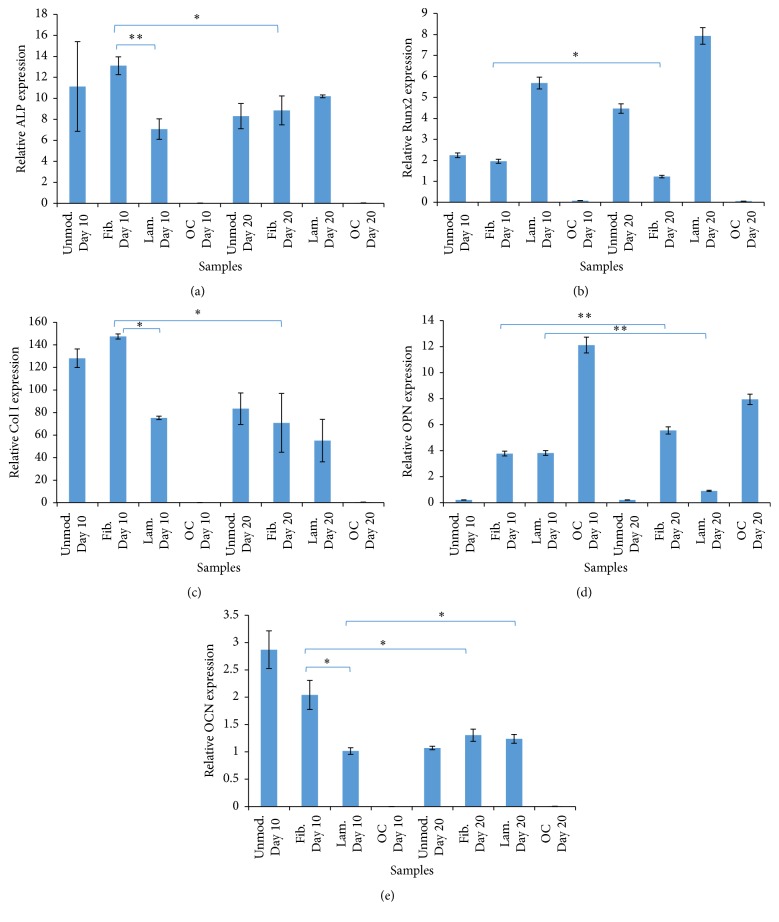
Expression levels of (a) alkaline phosphatase (ALP), (b) Runx2, (c) collagen type I (Col I), (d) osteopontin (OPN), and (e) osteocalcin (OCN) on fibronectin (fib.) or laminin (lam.) modified scaffolds, unmodified (unmod.) scaffolds, and TCP (OC refers to “only cell,” i.e., cells seeded on TCP) throughout 20 days of incubation (^*∗*^ *p* ≤ 0.1 and ^*∗∗*^ *p* ≤ 0.05).

Real-time PCR was performed to determine the expression levels of bone-specific genes: alkaline phosphatase (ALP), Runx2, collagen type I (Col I), osteopontin (OPN), and osteocalcin (OCN) (Figure 11). The PCR results were normalized using the housekeeping gene, beta-actin.

According to the results, the highest ALP production was significantly observed on the fibronectin modified scaffold at day 10 whereas laminin modified scaffold showed lower expression level of ALP (^*∗∗*^ *p* ≤ 0.05). As expected, significant decrease of the level of ALP expression on fibronectin modified scaffold was observed at day 20 (^*∗*^ *p* ≤ 0.1). Moreover, expression of Runx2 was significantly decreased from day 10 to day 20 on fibronectin modified scaffold (^*∗*^ *p* ≤ 0.1). However, on laminin modified scaffold, increase of the expression of Runx2 was observed throughout 20 days of incubation. Besides, collagen type I expression was significantly the highest on fibronectin and the lowest on laminin modified scaffolds at day 10. In fibronectin modified samples, at day 20, significant decrease in collagen type I level was observed (^*∗*^ *p* ≤ 0.1). Furthermore, although the expression level of osteopontin was similar on fibronectin and laminin modified scaffolds at day 10, it was significantly increased on fibronectin modified scaffold from day 10 to day 20 (^*∗∗*^ *p* ≤ 0.05) whereas decrease of osteopontin level was seen on laminin modified scaffold (^*∗∗*^ *p* ≤ 0.05). Finally, osteocalcin expression was observed to be significantly higher on fibronectin modified scaffold than on laminin modified scaffold at day 10. Throughout 20 days of incubation, the expression of osteocalcin decreased on fibronectin modified scaffold (^*∗*^ *p* ≤ 0.1).

## 5. Discussion

Designing a scaffold is an important part of tissue engineering and it requires a good knowledge of biomaterials and cell-surface interactions. A scaffold that is used in tissue engineering must be compatible with the selected cell type. Cell adherence to the substrate should be strong and maintained until the healthy tissue formation is observed.

In this study, polybutylene succinate (PBS) was used for the production of scaffolds by salt leaching technique due to good scaffolding properties of PBS, such as biocompatibility, biodegradability, nontoxicity, and porosity [2].

Pore size is a very important issue since pores are required for efficient flow of O_2_ and nutrients, cellular penetration, production of extracellular matrix, and neovascularization of the scaffold to achieve bone formation. It is well accepted that for bone tissue engineering purposes pore size should be within a range of 100–900 *μ*m [6]. In the present study, pore sizes of PBS scaffolds were measured as approximately 100 *μ*m which is within the accepted range.

In one of the studies, biodegradable textile-based structures were used for tissue engineering applications. For that purpose, silk fibroin (SF) filaments and polybutylene succinate (PBS) scaffolds were chosen. Although SF is a preferable scaffold for bone tissue engineering, SF matrices have low porosity because of their compact structures. To get the SF matrices with higher porosity and 100% interconnectivity, it can be used with PBS. PBS/SF construction can help us to observe porosity in desired ranges and get the minimal pore size (~75–100 *μ*m) required for bone tissue engineering studies. This study also proved that PBS scaffolds are suitable and usable to get desirable porosity of constructs [11].

The degradation behaviour of PBS scaffolds was also investigated in this study since it affects cell behaviour and tissue regeneration. The degradation rate is a balance between scaffold degradation and tissue regeneration [12]. For that reason, pH was measured at the end of 7, 15, 30, 60, and 120 days of incubation since the degradation products of PBS are butyric acid and succinic acid which could make the environment acidic enough to disturb the cells seeded on the scaffolds. During 120 days of incubation, pH was generally stable and its decrease was not in a level that would disturb the cells since the cells were not exposed to too much acidic environment by the degradation products of PBS and increase of cell proliferation was observed throughout 10 days of incubation in MTS assay. Wuertz et al. also studied the effect of pH on MSC proliferation. They showed that cell viability decreased with acidity [13]. The degradation of PBS scaffolds was also studied with respect to weight loss during 120 days of incubation. According to the results, weight loss started after 7 days of incubation and polymer was completely degraded after 120 days of incubation.

Although PBS is biocompatible enough, either surface modification of scaffolds with extracellular matrix proteins or blending of scaffolds with some biomaterials can also be applied in order to improve bioactivity of scaffolds. Both of them are recommended to observe increase in bioactivity. In one study, bioactivity of PBS scaffolds was improved by blending PBS with chitosan to obtain better cell attachment and viability [14]. Similarly in our study, we also aimed to increase the bioactivity of the scaffolds by the surface modification with fibronectin or laminin.

Fibronectin and laminin that have a sequence of amino acid, arginine–glycine–aspartic acid (RGD), are some of the ECM proteins. Cells can bind easily to RGD sequence of fibronectin due to their surface receptors called integrin. The surface of scaffolds can be modified with these materials to support cell attachment, growth, and differentiation onto the surface of the scaffolds [15, 16].

In addition, laminin modified surfaces are generally preferred to be used for neuron cell attachment and neuronal differentiation [17, 18]. In our study, the usage of laminin modified surfaces was preferred to seed pDGSCs since these cells are known as cells of neural crest origin. Thus, it was thought that pDGSCs might preferably attach onto the laminin modified surface.

Dental germs are preferable cell source since they contain MSCs which have the ability to be isolated, expanded, and cryopreserved easily. These cells maintain their properties after long-term cryopreservation. Also, dental germ stem cells (DGSCs) have the ability to differentiate into osteo-, adipo-, and neurogenic cells, easily [9]. Domestic pigs were used as an experimental model in the present study. Thus, DGSCs were isolated from the domestic pigs instead of human because they have the anatomical, physiological, and metabolic similarities with humans.

In this study, after cell seeding of unmodified and fibronectin and laminin modified scaffolds, cell proliferation was investigated throughout 10 days of incubation. Cell proliferation rate was the highest on fibronectin modified scaffolds and the lowest on laminin modified scaffolds. It was found out that increase in cell number on fibronectin modified scaffolds was much better than the others. In one of the studies, cell attachment on fibronectin modified PVDF surface and the application of PVDF as biomaterial in bone contact were studied. This study showed improvement in osteoblast adhesion on fibronectin modified PVDF surfaces [4]. This study supports our findings and showed the increase in cell proliferation due to fibronectin coating. It was also reported that cells proliferate readily on surfaces coated with fibronectin but poorly on surfaces coated with laminin [19]. Our MTS result also supported this finding. In another study it was demonstrated that human osteoblasts prefer to adhere to fibronectin compared to collagen types I and IV and vitronectin. In contrast, they adhere weakly to laminin and collagen type V and do not adhere to collagen type III at all [20].

In order to observe cell differentiation onto modified and unmodified scaffolds, some experiments such as ALP, immunocytochemistry, von Kossa staining, CM-Dil staining, and real-time PCR were carried out. Alkaline phosphatase is an early marker that shows beginning of the osteoblastic activity of the cell during the bone formation. Besides, it is an enzyme that plays an important role in mineralization. In the alkaline phosphatase activity assay, intracellular ALP was exposed by bursting pDGSCs seeded on PBS scaffolds. It was found from this assay that the expression of ALP was increased in all samples throughout 10 days of incubation. Then, it was decreased from mid stage (day 10) to late stage (day 20) in all scaffolds. This trend was expected since ALP is an early marker of osteoblast differentiation. It was shown before that ALP activity decreases when mineralization starts [21]. One of the studies showed that either collagen type I or fibronectin treated surfaces demonstrated early onset of mineralization and the enhancement of bone matrix secretion. Unlike fibronectin treated surfaces, laminin treated surfaces exhibited the failure of both mineralization and the presence of bone formation [21]. Mathews et al.'s study also supported our findings.

In von Kossa study, when unmodified and modified scaffolds were compared, mineralization was found to be the highest on the fibronectin modified scaffolds throughout 20 days of incubation. This and previous experiments showed that pDGSCs adhered and proliferated on fibronectin modified scaffolds more than the others and then they committed differentiation into bone tissue through 20 days of incubation and bone minerals were observed on the surface. According to one of the studies, the von Kossa staining revealed high calcium deposits on fibronectin treated surfaces [21]. Laminin modified surfaces showed the lowest staining on day 21 of osteogenic induction. These findings also support our results.

When the cell seeded unmodified and modified samples were observed under the confocal microscope, it was observed that the cells that were attached and dispersed onto the surface of the unmodified and fibronectin and laminin modified foams showed decrease in their proliferation rate throughout 20 days of incubation due to differentiation, especially in fibronectin modified scaffolds. After the cells stained with Phalloidin and TO-PRO on PBS scaffolds, it was observed that the cell attachment and proliferation occurred especially around the pores of the foam. This might be due to the accessibility of oxygen and nutrients. Cell seeded modified and unmodified foams were also stained with collagen type I. It was observed that cells on all scaffolds synthesized collagen type I. This indicates the differentiation of cells to bone and the secretion of extracellular matrix demonstrated the initialization of tissue formation. Throughout 20 days of incubation, the amount of synthesized collagen was increased and found to be the highest, especially on the fibronectin modified scaffolds. It also proved that cell differentiation was better on fibronectin modified scaffolds compared to the others.

In one of the experiments, it was showed that the usage of fibronectin generally supplies benefits for cell attachment and spreading even if different techniques are applied for the attachment of fibronectin onto the surfaces [22]. According to the study, fibronectin was conjugated onto highly porous 3D poly(carbonate) urethane scaffolds to distribute cells rapidly throughout the scaffold. In another study, MSCs were seeded on modified poly(HEMA/MA) hydrogel surfaces and cultivated for 4 days. They were stained with Phalloidin and their fluorescence microscopy images were taken. According to the results of this experiment, cells adhered poorly and did not proliferate on unmodified poly(HEMA/MA) hydrogel. Cell growth increased after collagen I coating of hydrogel surface. However, cell growth significantly increased by the attachment of fibronectin and laminin on the collagen layer, especially with fibronectin attachment [23].

Cell-to-cell and cell-to-matrix interaction were also determined by CM-Dil staining in our study. Cellular spreading could be observed because of the binding of a thiol-reactive chloromethyl moiety in the structure of CM-Dil dye to cellular thiols. According to results, increase in cell number was observed on unmodified PBS scaffold throughout 7 days of incubation. On the other hand, cell number was decreased on fibronectin or laminin modified scaffolds after 7 days of incubation. It might be due to cell differentiation on modified PBS scaffolds. This data also supports confocal microscopy results.

Finally, the expression of bone-specific genes which were ALP, Runx2, collagen type I, osteopontin, and osteocalcin was analyzed by real-time PCR. According to the results, the highest ALP expression was observed on fibronectin modified scaffolds at day 10. Then, there was a decrease in ALP expression at day 20. However, ALP expression on laminin modified scaffold was increased throughout 20 days of incubation period. This result was expected due to the increase of ALP enzyme activity in the early stages of the cell culture and this elevated ALP activity was assumed to show the number of osteogenic committed progenitor cells in the culture [24].

During bone formation, lots of transcription factors are needed for the activation of osteoblast differentiation. One of them is Runx2 that is expressed at early stages of differentiation [25]. In our results, decrease of the expression of Runx2 was observed throughout 20 days of incubation on fibronectin modified scaffold since it was induced at early stages whereas Runx2 expression on laminin modified scaffold was increased throughout 20 days of incubation period. Additionally, the direction of preosteoblast cells into immature osteoblasts can be provided by Runx2 which then binds to promoter regions of bone-specific genes such as collagen type I (Col I), alkaline phosphatase (ALP), osteocalcin (OCN), and osteopontin (OPN) and activates their expression. However, expression levels of these genes can differ according to the osteoblast maturation stages. The expression of Col I and OPN proteins is provided by immature osteoblasts, while OCN protein is strongly expressed by early and late mature osteoblasts. Although bone matrix proteins are expressed by immature osteoblasts, the cells are unable to induce bone mineralization [26].

According to the results, the expression of collagen type I that was the major part of the organic extracellular matrix was supplied by immature osteoblasts as the highest one at day 10 on fibronectin modified scaffold. It showed that rapid bone formation was more on fibronectin modified scaffold than on laminin modified and unmodified scaffold since the organic matrix of bone was comprised of collagen which was synthesized by osteoblasts. At day 20, decrease in expression level of collagen was observed due to the bone remodeling cycle where the osteoblast recruitment potential of the collagen is demineralized by the osteoclast. After that, it triggers bone matrix formation through the osteoblasts that subsequently become mineralized [27, 28].

At the initial stage of bone formation, the production of ALP and Col I is observed by osteoblasts [29]. These proteins supply extracellular matrices suitable enough for mineral deposition. After that, the expressions of osteopontin and osteocalcin proteins are seen. These are also related to bone-mineral deposition.

Additionally, expressions of osteopontin and osteocalcin were analyzed. Osteopontin was a multifunctional phosphorylated glycoprotein secreted by osteoblasts [30]. The increase of the osteopontin expression was seen from day 10 to day 20 on fibronectin modified scaffold since through the mineralization phase osteopontin expression could be seen at maximum level [29]. It showed that bone formation occurred during 20 days of incubation. However, decrease of the osteopontin expression was observed through the mineralization phase on laminin modified scaffold. Also, osteocalcin was highly expressed on fibronectin modified scaffold at day 10 compared to laminin modified scaffold whereas decrease of their expression was observed on fibronectin modified scaffold at day 20. Osteocalcin could be expressed by early and late mature osteoblasts [26] and the secretion of bone matrix protein “osteocalcin” could be seen at maximum level through the mineralization phase, like osteopontin [29]. Therefore, our results could be due to the presence of mineralized bone matrix that was obtained on fibronectin modified scaffold at day 10. It also showed that the mesenchymal stem cells differentiated into mature osteoblasts on fibronectin modified scaffold more than the others.

## 6. Conclusion

This study aimed to observe the effect of fibronectin or laminin modified polybutylene succinate (PBS) scaffolds on porcine dental germ stem cell (pDGSC) differentiation into bone for the treatment of injuries of the critical size bone defects.

PBS scaffolds were preferred due to their appropriate properties for cell attachment, alignment, and differentiation. Surface modification of PBS scaffolds was made by using either fibronectin or laminin to provide better cell proliferation and to develop surface biocompatibility. According to the results, the surface modification of PBS scaffolds with fibronectin modification can be preferable for pDGSCs, compared to the surface modification with laminin.

In conclusion, all tests showed the positive effects of fibronectin and laminin modifications on PBS scaffolds for proliferation and differentiation of pDGSC into bone. PBS scaffold has a good potential for the healing of critical size bone defect. Besides, pDGSCs have a great potential for bone tissue engineering studies.* In vivo* experimentation should be carried out to show the success of this material for bone tissue engineering studies.

## Figures and Tables

**Table 1:** Sequences of the primers used for the real-time PCR.

ALP	F: CGACAACTACCAGGCACAGT	Tm: 57°C	Product length: 248
	R: GCCCTCAGAACAAGATGCCT		

Runx2	F: ACTGAACCCCACGCTTGTTC	Tm: 59°C	Product length: 253
	R: AGTCACCTCCGCTTTCAAGG		

Osteopontin	F: AGTCCAACGAAAGCCCTGAG	Tm: 57°C	Product length: 292
	R: GCTTCGGATCTGCGGAACTT		

Osteocalcin	F: CCTAGTGGTGCGGATTCTGG	Tm: 55°C	Product length: 241
	R: GCTGCGAGGTCTAGGCTATG		

Collagen type I	F: GACATCCCACCAGTCACCTG	Tm: 58°C	Product length: 229
	R: CTCCCGTGGTTTCCTGGTC		

Beta-actin	F: GACTTCGAGCAGGAGATGG	Tm: 56°C	Product length: 233
	R: GCACCGTGTTGGCGTAGAG		

**Table 2:** Cell surface antigen expression of pDGSCs.

Surface antigen	Percentage of positively marked pDGSCs
pDGSCs (only cells)	1.27
CD105	88.71
CD90	99.04
CD44	98.23
CD45	3.44
CD34	1.18
